# Transcription factors in tanshinones: Emerging mechanisms of transcriptional regulation

**DOI:** 10.1097/MD.0000000000040343

**Published:** 2024-11-22

**Authors:** Yanyun Pan, Jin Dai, Minwei Jin, Qiujun Zhou, Xiaoliang Jin, Jinjie Zhang

**Affiliations:** a Department of Cardiology, The First Affiliated Hospital of Zhejiang Chinese Medical University (Zhejiang Provincial Hospital of Chinese Medicine), Hangzhou, Zhejiang, China; b Department of Cardiology, The First Affiliated Hospital of Zhejiang Chinese Medical University (Zhejiang Provincial Hospital of Chinese Medicine), Hangzhou, Zhejiang, China; c Department of Orthopaedics, The First Affiliated Hospital of Zhejiang Chinese Medical University (Zhejiang Provincial Hospital of Chinese Medicine), Hangzhou, Zhejiang, China; d The First School of Clinical Medicine, Zhejiang Chinese Medical University, Hangzhou, Zhejiang, China; e The First School of Clinical Medicine, Zhejiang Chinese Medical University, Hangzhou, Zhejiang, China; f Department of Orthopaedics, The First Affiliated Hospital of Zhejiang Chinese Medical University (Zhejiang Provincial Hospital of Chinese Medicine), Hangzhou, Zhejiang, China.

**Keywords:** tanshinones, TFs, Traditional Chinese Medicine, transcription factor, transcriptional regulation

## Abstract

Transcription factors play a crucial role in the biosynthesis of tanshinones, which are significant secondary metabolites derived from *Salvia miltiorrhiza*, commonly known as Danshen. These compounds have extensive pharmacological properties, including anti-inflammatory and cardioprotective effects. This review delves into the roles of various transcription factor families, such as APETALA2/ethylene response factor, basic helix-loop-helix, myeloblastosis, basic leucine zipper, and WRKY domain-binding protein, in regulating the biosynthetic pathways of tanshinones. We discuss the emerging mechanisms by which these transcription factors influence the synthesis of tanshinones, both positively and negatively, by directly regulating gene expression or forming complex regulatory networks. Additionally, the review highlights the potential applications of these insights in enhancing tanshinone production through genetic and metabolic engineering, setting the stage for future advancements in medicinal plant research.

## 1. Introduction

Tanshinones are a group of diterpenoid quinones primarily extracted from the roots of *Salvia miltiorrhiza*, an herb widely employed in Traditional Chinese Medicine.^[1]^ These compounds are celebrated for their diverse pharmacological properties, which include cardiovascular protection, antioxidation, and anti-inflammatory effects, making them of great interest in both pharmacological research and clinical applications.^[2,3]^ In recent years, tanshinones have garnered attention for their potential therapeutic applications in treating a range of diseases, including cancer, cardiovascular diseases, and neurodegenerative disorders. The ability of tanshinones to modulate various biological pathways positions them as promising candidates for the development of new therapeutic agents. Furthermore, their anti-inflammatory and antioxidant properties are being explored in the context of chronic inflammatory diseases and conditions characterized by oxidative stress.^[4]^

The biosynthesis of tanshinones involves intricate pathways that are tightly regulated at various levels, one of the most significant being transcriptional regulation. Transcription factors (TFs) play a crucial role in this process, acting as molecular switches that can either initiate or repress the expression of genes involved in tanshinone biosynthesis.^[5]^ These factors respond to internal and external stimuli, ensuring that the production of tanshinones is adaptive to the plant’s developmental needs and environmental stresses. Research into the transcriptional regulation of tanshinone biosynthesis has highlighted several key TF families, including APETALA2/ethylene response factor (AP2/ERF), basic helix-loop-helix (bHLH), myeloblastosis (MYB), basic leucine zipper (bZIP), and WRKY domain-binding protein (WRKY).^[6]^ These TFs interact with specific promoter regions of genes encoding enzymes critical to the biosynthetic pathways, such as those leading to the formation of diterpene compounds, which are precursors to tanshinones.^[7]^

Understanding the roles and mechanisms of these TFs is not only fundamental for elucidating the regulatory networks of secondary metabolite biosynthesis but also essential for strategies aimed at enhancing the production of these compounds. Techniques such as overexpression, silencing, and clustered regularly interspaced short palindromic repeats/CRISPR-associated protein 9-mediated gene editing are being applied to modify the activity of specific TFs, thereby adjusting the flux through the biosynthetic pathways to increase the yield of tanshinones.^[8]^ Moreover, the study of TFs in *S miltiorrhiza* provides insights into the broader field of plant biology, offering clues about how plants control the production of secondary metabolites, which are crucial for their survival, reproduction, and resilience against environmental challenges.^[9]^

This introduction sets the stage for a detailed review of the specific TFs involved in the biosynthesis of tanshinones, discussing their roles, regulation, and the potential applications of this knowledge in biotechnological approaches to enhance the production of these valuable medicinal compounds.

## 2. Biosynthetic pathways of tanshinone compounds

**Figure 1.**
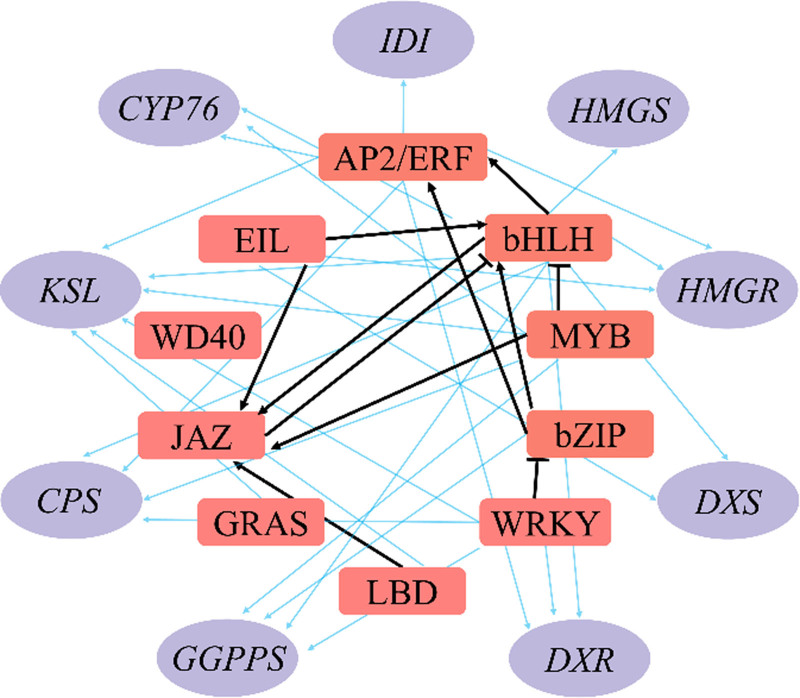
The transcription factor-enzyme gene regulation network. In this diagram, the orange squares denote transcription factors, while the purple ovals depict key enzyme genes. Blue arrows represent the interactions between transcription factors and key enzyme genes, highlighting the regulatory connections. Black arrows show the enhancement of gene expression by transcription factors, whereas black T-lines represent the suppression or inhibition mediated by these transcription factors. This visual encapsulates the complex regulatory interactions that orchestrate gene expression within the cell. AP2/ERF = APETALA2/ethylene response factor, bHLH = basic helix-loop-helix, bZIP = basic leucine zipper, CPS = copalyl diphosphate synthase, CYP76 = cytochrome P450 76, DXR = 1-deoxy-D-xylulose 5-phosphate reductoisomerase, DXS = 1-deoxy-D-xylulose 5-phosphate synthase, EIL = Ethylene-insensitive3-like, GGPP = geranylgeranyl diphosphate, GRAS = GAI-RGA-and-SCR, HMGR = 3-hydroxy-3-methylglutaryl-CoA reductase, HMGS = 3-hydroxy-3-methylglutaryl-CoA synthase, IDI = Isopentenyl-Diphosphate Delta Isomerase, JAS = jasmonates, KSL = Kaurene Synthase-like, LBD = lateral organ boundaries domain, MYB = myeloblastosis, WRKY = WRKY domain-binding protein.

Tanshinone biosynthesis begins with the formation of essential precursors, isopentenyl pyrophosphate and its isomer, dimethylallyl pyrophosphate. These precursors are synthesized through 2 distinct pathways: the mevalonate (MVA) pathway located in the cytoplasm, and the 2-C-methyl-D-erythritol 4-phosphate (MEP) pathway situated in the plastids (Fig. 1).^[10,11]^

The key enzymes in the MVA pathway include acetoacetyl-CoA thiolase, 3-hydroxy-3-methylglutaryl-CoA synthase, and 3-hydroxy-3-methylglutaryl-CoA reductase (HMGR), while the MEP pathway involves critical enzymes such as 1-deoxy-D-xylulose 5-phosphate synthase (DXS), 1-deoxy-D-xylulose 5-phosphate reductoisomerase (DXR), and 1-hydroxy-2-methyl-2-(E)-butenyl-4-diphosphate reductase. HMGR serves as the first rate-limiting enzyme in the MVA pathway, with multiple forms such as HMGR1, HMGR2, HMGR3, and HMGR4 identified in the *S miltiorrhiza* genome. Overexpression of the SmHMGR2 gene has been shown to increase tanshinone content.^[12,13]^ DXS is identified as the first rate-limiting enzyme in the MEP pathway, with 5 DXS genes noted in the *S miltiorrhiza* genome. SmDXS2 is particularly notable for its higher expression level in the roots, and its overexpression has been linked to increased tanshinone content.^[13–15]^

Downstream in the pathway, the diterpene precursor geranylgeranyl diphosphate (GGPP) is synthesized by GGPPS catalyzing isopentenyl pyrophosphate and dimethylallyl pyrophosphate. Variations in TF binding sites within the promoters of SmGGPPS1 and SmGGPPS3 have been significantly correlated with the content of tanshinone components. Additionally, the accumulation of tanshinones is positively associated with the expression of the key enzyme genes SmCPS and SmKSL. Enzymes such as CYP76AH1, CYP76AH3, and CYP76AK1 are involved in the structural modification of tanshinone compounds. Under the catalytic influence of CYP76AH1, tanshinone diene is converted into ferruginol, which is further transformed by CYP76AH3 and CYP76AK1 into 11,20-dihydroxy ferruginol and 11,20-dihydroxy sugiol.^[16–20]^ Ultimately, the synthesis of tanshinone components is mediated by the CYP450 family, although the specific steps of these catalytic reactions remain to be further elucidated and require additional research.

## 3. TFs regulating the biosynthesis of tanshinone components

TFs play a critical role as regulators within the network of secondary metabolite synthesis. They regulate the synthesis of tanshinones primarily in 2 ways. First, TFs can activate or inhibit the expression of 1 or several genes within the tanshinone biosynthetic pathway, thereby positively or negatively influencing tanshinone biosynthesis. For example, SmERF73 positively regulates tanshinone synthesis by activating the expression of the DXR1, CPS1, KSL1, and CYP76AH3 genes.^[21]^ Conversely, SmMYB4 negatively regulates tanshinone synthesis by activating the expression of the GGPPS3 gene.^[22]^ Second, individual TFs can interact with other TFs to form complexes that regulate target gene expression. For instance, the jasmonate ZIM-domain (JAZ) protein interacts with SmbHLH59 or SmMYB97 to inhibit the expression of target genes, thereby suppressing tanshinone synthesis.^[23]^ Several families of TFs have been reported to participate in the regulation of tanshinone component biosynthesis, including AP2/ERF, bHLH, MYB, bZIP, and WRKY.

### 3.1. Tissue specificity of TFs

In *S miltiorrhiza*, the redness of the periderm is preferred, where the redder it is, the higher the content of tanshinones, which are the red secondary metabolites especially concentrated in the peridermal cork layer.^[1]^ Current studies show that the expression of the GAI-RGA-and-SCR (GRAS) TF is significantly higher in the root periderm than in other parts, indicating that GRAS plays an important role in the regulatory synthesis of tanshinones.^[24]^ Other types of TFs are also predominantly expressed in the roots of *S miltiorrhiza*, such as those belonging to the ERF subfamily, including SmERF73,^[21]^ SmERF72,^[25]^ and SmAP2/ERF82,^[26]^ which have the highest expression in the roots. Therefore, identifying TFs with tissue-specific expression could reveal their roles in the synthesis and accumulation of tanshinones.

### 3.2. Regulation of TFs by inducers

TF families such as AP2/ERF, bHLH, MYB, bZIP, and WRKY are induced by elicitors like methyl jasmonate (MeJA), salicylic acid, abscisic acid, gibberellins (GA), jasmonic acid (JA), and yeast extract, demonstrating their capability to regulate tanshinone synthesis. For example, external application of MeJA significantly induces or suppresses the expression of SmJAZ in hairy roots.^[27]^ As a key regulatory factor in the GA signaling pathway, the expression of GRAS TFs is significantly upregulated or downregulated following GA induction.^[24]^ Thus, a systematic analysis of the expression patterns of TF families induced by these elicitors can help understand their regulatory capabilities on tanshinone synthesis.

### 3.3. The regulatory role of TFs in tanshinone synthesis

In this section, we provide a detailed introduction to the TFs that influence the synthesis of tanshinones, summarizing those that have either positive or negative effects on their biosynthetic pathway and their specific target genes (Table 1).

**Table 1:** The regulatory role of transcription factors in tanshinone synthesis.

Family	Member	Target gene	Reference
AP2/ERF	SmERF72	HMGR, CYP76AH3, KSL1, ID1	^[28]^
	SmERF98	CYP76AH3, KSL1, IDI1	^[28]^
	SmERF73	DXR1, CPS1, KSL1, CYP76AH3	^[23]^
	SmERF128	CYP76AH1, CPS1, KSL1	^[29]^
	SmERFlL1	DXR	^[30]^
	SmERF2	CPS1	^[31]^
	SmERF6	KSL1, CPS1	^[32]^
	SmERF8	KSL1	^[32]^
	SmAP2/ERF82	IDI1, CPS1, CYP76AH3	^[26]^
	SmERFl	/	^[33]^
bHLH	SmbHLH61	/	^[34]^
	SmbHLH74	HMGRl, GGPPS1, CYP76AH1	^[35]^
	SmbHLH92	DXR, HMGR4	^[36]^
	SmbHLH7	DXS2, CPS1, KSL1, CYP76AH1	^[37]^
	SmbHLH10	DXS2, CPS1, CPS5	^[37]^
	SmbHLH130	CPS1, DXS2, KSL1, CYP76AH1	^[37]^
	SmbHLH3	KSL1, CYP76AH1	^[37]^
	SmbHLH148	DXS2, CPS1, CYP76AH1	^[37]^
	SmbHLH59	CPS1, KSL1	^[23]^
MYB	SmMYB9b	/	^[38]^
	SmMYB98	GGPPS1	^[39]^
	SmMYB1	/	^[40]^
	SmMYB36	HMGS1, GGPPS, DXR, CMK, MCT	^[41]^
	SmMYB4	/	^[42]^
	SmMYB39	/	^[37]^
	SmMYB97	CPS1, KSL1	^[43]^
bZIP	SmbZIP1	GGPPS	^[44]^
	SmbZIP3	/	^[45]^
	SmHY5	/	^[46]^
WRKY	SmWRKY1	DXR	^[47]^
	SmWRKY2	CPS	^[48]^
	SmWRKY14	CPS1, DXS2, KSL1, CYP76AH1	^[23]^
	SmWRKY44	CPS1, CPS5, KSL1	^[49]^
	SmWRKY40	CPS1, CPS5	^[50]^
	SmWRKY34	GGPPS	^[45]^
	SmWRKY54	KSL	^[51]^
	SmWRKY61	/	^[52]^
LBD	SmLBD44	KSL1	^[53]^
GRAS	SmGRAS1	KSLl	^[54]^
	SmGRAS2	/	^[54]^
	SmGRAS3	KSLl	^[54]^
	SmGRAS4	KSLl	^[54]^
	SmGRAS5	KSLI	^[54]^
JAZ	SmJAZ1	/	^[27]^
	SmJAZ2	/	^[27]^
	SmJAZ5	/	^[27]^
	SmJAZ6	/	^[27]^
	SmJAZ9	/	^[27]^
	SmJAZ3	/	^[27]^
	SmJAZ4	/	^[27]^
	SmJAZ8	/	^[27]^
WD40	SmWD40-170	/	^[55]^
EIL	SmEIN3	HMGR, DXS2	^[56]^

AP2/ERF = APETALA2/ethylene response factor, bHLH = basic helix-loop-helix, bZIP = basic leucine zipper, DXR = 1-deoxy-D-xylulose 5-phosphate reductoisomerase, DXS = 1-deoxy-D-xylulose 5-phosphate synthase, EIL = Ethylene-insensitive3-like, GGPP = geranylgeranyl diphosphate, GRAS = GAI-RGA-and-SCR, HMGR = 3-hydroxy-3-methylglutaryl-CoA reductase, HMGS = 3-hydroxy-3-methylglutaryl-CoA synthase, JAZ = jasmonate ZIM-domain, LBD = lateral organ boundaries domain, MYB = myeloblastosis, WRKY = WRKY domain-binding protein.

#### 3.3.1. AP2/ERF transcription factors

AP2/ERF TFs, forming one of the largest families in plant transcription, possess an AP2 domain composed of about 60 amino acids that can directly interact with cis-elements. These factors are classified into 5 subfamilies based on the number of AP2 domains and their binding sequences: AP2, ERF, DREB, RAV, and Soloist. The ERF subfamily, responsive to ethylene signaling pathways, has been extensively explored for its regulatory role in tanshinone synthesis. Research by Ji et al^[57]^ has identified 179 AP2/ERF TFs within the *S miltiorrhiza* genome, categorized into 5 subfamilies based on the characteristics of the AP2 domain. Their study, integrating gene expression analysis, co-expression networks, and cis-regulatory elements, suggests that SmERF128 and SmERF152 are pivotal in regulating tanshinone biosynthesis.

Current studies highlight that ERF TFs generally promote tanshinone synthesis. Notably, SmERF72 and SmERF98 engage with the Guanylate cyclization C (GCC)-box in the promoters of target genes such as HMGR, KSL1, and CYP76AH3, enhancing gene transcription.^[28]^ Similarly, SmERF73 interacts with the GCC-box in the promoters of DXR1, CPS1, KSL1, and CYP76AH3, activating these genes.^[22]^ SmERF128 binds to GCC-box, C-repeat-binding transcription factor 2, and Recombinase Aided Amplification motifs, thus upregulating SmCPS1, SmKSL1, and SmCYP76AH1, which boosts tanshinone production.^[30]^ Additionally, the subcellular localization assay revealed that SmERF1L1 is localized in the nucleus. Overexpression of SmERF1L1 in transgenic *S miltiorrhiza* hairy roots led to a significant increase in tanshinone production by broadly upregulating genes involved in the tanshinone biosynthetic pathway, with SmDXR being particularly enhanced. Yeast 1-hybrid and electrophoretic mobility shift assays confirmed that SmERF1L1 binds to the GCC-box in the SmDXR promoter, while the dual-luciferase (Dual-LUC) assay demonstrated that SmERF1L1 positively regulates SmDXR expression,^[31]^ and overexpression of SmERF2 significantly increases tanshinone levels, indicating its positive role in the biosynthetic process.^[31]^

Moreover, ERF TFs are crucial not only in enhancing tanshinone synthesis but also in supporting growth development and abiotic stress responses. For instance, SmERF6 regulates tanshinone biosynthesis by directly binding to the ethylene-responsive elements (GCC-box) in the promoters of SmKSL1 and SmCPS1, thereby activating their transcription. Additionally, the accumulation of tanshinones helps maintain the homeostasis of total phenolic acids and flavonoids in *S miltiorrhiza*. These findings elucidate how SmERF6 directly co-regulates the transcription of SmCPS1 and SmKSL1, thereby modulating tanshinone synthesis and accelerating the metabolic flux for tanshinone accumulation in *S miltiorrhiza*.^[32]^ SmAP2/ERF82, by regulating key enzymes like IDI1, CPS1, and CYP76AH3, positively affects tanshinone biosynthesis. Contrarily, overexpression leads to stunted growth, while RNAi lines display robust growth due to the negative regulation of GA biosynthesis by SmAP2/ERF82.^[26]^ Additionally, SmERF1, while less effective in tanshinone regulation, enhances the salt tolerance of *S miltiorrhiza*.^[33]^

#### 3.3.2. bHLH transcription factors

The bHLH family, one of the largest families of eukaryotic TFs, features a highly conserved basic/helix-loop-helix domain composed of about 50 to 60 amino acids. This domain is divided into 2 parts: the basic amino acid region at the N-terminus, which specifically binds to the DNA sequences in the promoters of target genes, and the helix-loop-helix region at the C-terminus, which promotes protein-protein interactions and forms homodimers or heterodimers to control gene transcription.^[58,59]^ Zhang et al^[60]^ identified 127 bHLH TFs in the *S miltiorrhiza* genome, classified into 25 subfamilies based on phylogenetic analysis. After treatment with MeJA, 7 bHLH genes (SmbHLH37/51/53/60/74/92/103) were found to potentially participate in the regulation of tanshinone metabolism.

bHLH TFs participate in both positive and negative regulation of tanshinone component synthesis. Initially, SmbHLH61,^[34]^ SmbHLH7,^[37]^ SmbHLH10,^[37]^ SmbHLH130,^[37]^ SmbHLH148,^[37]^ and SmbHLH59^[23]^ are involved in the positive regulation of tanshinones. Xing et al^[37]^ discovered that SmbHLH7, SmbHLH10, SmbHLH130, and SmbHLH148 regulate gene transcription by binding to the E/G-box elements in the promoters of key enzyme genes in the tanshinone biosynthesis pathway. SmbHLH59 activates the expression of CPS1 and KSL1 by binding to the E/G-box elements in the promoter region.^[23]^ Conversely, SmbHLH3^[37]^ and SmbHLH74^[35]^ negatively regulate tanshinone synthesis. SmbHLH3 binds to elements in the promoters of KSL1 and CYP76AH1, inhibiting the metabolism of tanshinone compounds,^[37]^ while SmbHLH74 directly inhibits the transcription of SmHMGR1, SmGGPPS1, and SmCYP76AH1, negatively regulating tanshinone biosynthesis.^[35]^ Additionally, SmbHLH92 has a dual regulatory effect on tanshinone accumulation, negatively regulating dihydrotanshinone I and cryptotanshinone and positively regulating tanshinone I and tanshinone IIA. Further transcriptome data suggest that SmbHLH92 may regulate gene expression by directly binding to the G-box in the promoters of SmHMGR4, SmGPPS.LSU, SmGPPS.SUII.2, and SmDXR.^[36]^

bHLH TFs are regulated by other TFs, dynamically modulating the synthesis of tanshinone components. For example, SmMYC2 directly inhibits the transcription of SmbHLH74, weakening the inhibitory effect of SmbHLH74 on tanshinone accumulation.^[35]^ SmJAZ1/8 proteins inhibit the transcriptional activity of SmbHLH59, thereby inhibiting tanshinone biosynthesis.^[23]^ SmbHLH7 and SmMYB39 regulate the target genes of tanshinone metabolism and compete for binding to these genes. When they form a complex, SmbHLH7 prevents other positively regulating MYB TFs from forming complexes, thus jointly regulating the dynamic balance of secondary metabolism in *S miltiorrhiza*.^[37]^

#### 3.3.3. MYB transcription factors

The MYB family of TFs is large and diverse, playing crucial roles in plant growth and development. The N-terminus of MYB proteins features a highly conserved DNA-binding domain with 1 to 3 repeat sequences (R1, R2, and R3), each composed of 51 to 52 amino acids that fold into 3 α-helices, with the second and third helices forming a helix-turn-helix structure. Based on the number and position of structural domains, the MYB family is divided into 1R (R1/2, R3-MYB), 2R (R2R3-MYB), 3R (R1R2R3-MYB), and 4R (R1R2R2R1/2-MYB), with most MYB proteins belonging to the R2R3-MYB subfamily.^[61,62]^ Li et al^[63]^ identified and analyzed 110 R2R3-MYB TFs from the *S miltiorrhiza* genome. Based on phylogenetic analysis and research on Arabidopsis, they were divided into 37 subgroups, predicting that members of subgroups 4, 5, and 20 are potential regulators of terpenoid synthesis in *S miltiorrhiza*.

MYB TFs not only positively regulate the accumulation of tanshinones but also affect the development of the *S miltiorrhiza* root system, such as SmMYB9b,^[38]^ SmMYB98,^[39]^ and SmMYB1.^[40]^ SmMYB9b activates the transcription levels of genes related to the MEP pathway in *S miltiorrhiza*, including SmDXS2, SmDXR, SmGGPPS, and SmKSL1, exerting a positive regulatory effect. Overexpression of SmMYB9b results in finer, fewer lateral roots and is more prone to forming callus tissue and differentiating into buds compared to the control group.^[38]^ Overexpression of SmMYB98 leads to shorter and thicker hairy roots compared to the control group, with reduced GA content and increased tanshinone content, indicating that SmMYB98 affects the growth of *S miltiorrhiza* hairy roots, negatively regulating GA biosynthesis and positively regulating tanshinone synthesis.^[39]^ SmMYB1, as an important feedback regulator, promotes the growth of *S miltiorrhiza* hairy roots and the accumulation of tanshinone compounds in the hairy roots.^[40]^ SmMYB36 enhances tanshinone accumulation by increasing the transcription levels of DSX1, DXS2, DXR, MCT, MDS, HDS, CMK, 1-hydroxy-2-methyl-2-(E)-butenyl-4-diphosphate reductase 1, GGPPS1, CPS1, CYP76AH1, and KSL1.^[41]^ MYB TFs can also negatively regulate tanshinone synthesis. Compared to the control, overexpression of SmMYB4 leads to decreased levels of cryptotanshinone and tanshinone IIA, while interference with SmMYB4 results in increased levels of these compounds. The expression of the key enzyme gene GGPPS3 in the tanshinone biosynthesis pathway is significantly affected, suggesting that SmMYB4 negatively regulates tanshinone synthesis by controlling GGPPS3.^[42]^

Additionally, MYB TFs form MBW protein complexes with bHLH and WD40 proteins to jointly regulate secondary metabolism. Xing et al^[37]^ found that overexpression of SmMYB39 suppresses the content of tanshinone compounds and the expression of genes in the tanshinone biosynthesis pathway, including DXS2, DXR, HMGR1, GGPPS, and KSL1, in hairy roots. Interaction between SmMYB39 and SmbHLH7 promotes the transcription of SmMYB39 when SmbHLH7 is overexpressed, while silencing SmbHLH7 inhibits the transcription of SmMYB39, with the 2 factors jointly regulating the dynamic balance of tanshinone accumulation. Li et al^[43]^ found that overexpression of SmMYB97 increases tanshinone content and upregulates the expression of genes in the tanshinone synthesis pathway, including SmDXS1, SmHMGR1, SmFPPS, SmGPPS, SmGGPPS, SmCPS1, SmKSL1, and SmCYP76AH1. Yeast 1-hybrid and transient transcriptional activity assays showed that SmMYB97 binds to the promoter regions of CPS1 and KSL1, activating their expression. Yeast 2-hybrid and bimolecular fluorescence complementation experiments showed that SmMYB97 interacts with SmJAZ8, jointly inhibiting the expression of SmCPS1 and SmKSL1, indicating that SmMYB97 and SmJAZ8 participate in the process of tanshinone accumulation.

#### 3.3.4. bZIP transcription factors

The bZIP family of TFs is large and diverse, containing a bZIP domain composed of 60 to 80 amino acids. The N-terminal region is relatively conserved, containing a nuclear localization signal and an N-X7-R/K motif; the C-terminal region is a leucine zipper area, composed of leucine or other hydrophobic amino acids, which can form homodimers or heterodimers to function.^[64,65]^ Zhang et al^[66]^ conducted the first whole-genome analysis of the bZIP gene family in *S miltiorrhiza*, identifying 70 SmbZIP TFs, divided into 11 subgroups based on phylogenetic relationships with Arabidopsis. SmbZIP7 and SmbZIP20 are likely involved in the regulation of tanshinone biosynthesis.

bZIP TFs directly or indirectly affect the biosynthesis of tanshinones through different pathways. For example, after abscisic acid treatment of *S miltiorrhiza* hairy roots, bZIP3/6/10/18/19/36/37/68/71 are significantly related to 1 or more key enzyme genes in the tanshinone biosynthesis pathway, suggesting that bZIP TFs may regulate the biosynthesis of active components in *S miltiorrhiza* by binding to gene promoters in the tanshinone biosynthesis pathway and activating gene transcription.^[67]^ SmbZIP1 binds to the G-Box element, directly inhibiting the expression of the GGPPS gene and reducing tanshinone content.^[44]^ SmbZIP3 indirectly promotes tanshinone biosynthesis by regulating the interaction between TFs SmERF128 and SmMYB9b.^[45]^ Additionally, SmHY5 has multiple regulatory effects on the morphological development of *S miltiorrhiza* roots and the accumulation of active components. Overexpression of SmHY5 reduces the number of primary and secondary lateral roots and fresh weight of *S miltiorrhiza*, increases the diameter and length of primary lateral roots, and enhances tanshinone content, while silencing the SmHY5 gene results in the opposite effects.^[46]^

#### 3.3.5. WRKY transcription factors

The WRKY family is one of the largest families of TFs in higher plants, with its DNA-binding domain containing approximately 1 or 2 WRKY structures of 60 amino acids each. The N-terminal region contains a conserved heptapeptide sequence WRKYGQK, and the C-terminal region has a C2H2 or C2HC zinc finger structure.^[68]^ WRKY proteins function by specifically binding to the W-box in the promoters of target genes, with the specific sequence of the W-box being (C/T)TGAC(C/T) and TGAC being its core sequence.^[69]^ Li et al^[70,71]^ identified 61 SmWRKY TFs from the *S miltiorrhiza* genome, with multiple sequence alignments indicating that SmWRKY can be divided into 3 classes, with 42 responding to yeast extract and Ag+ stress, suggesting that SmWRKY1/3/7/9/12/19/25/29/30/35/42/52/56/58/63/68 are involved in tanshinone synthesis.

WRKY TFs regulate target gene transcription by binding to W-box elements in the promoters of key enzyme-encoding genes in the tanshinone synthesis pathway, such as SmWRKY1,^[47]^ SmWRKY2,^[48]^ SmWRKY14,^[23]^ SmWRKY44,^[49]^ and SmWRKY40.^[50]^ Cao et al^[47]^ found that overexpression of SmWRKY1 significantly enhances the expression of key enzyme genes in the MEP pathway, particularly SmDXR, with dual-luciferase reporter assays proving that SmWRKY1 directly binds to the promoter region and transcriptionally activates SmDXR expression, positively regulating tanshinone biosynthesis. SmWRKY2 binds to SmCPS,^[48]^ SmWRKY14 binds to SmCPS1,^[23]^ SmWRKY44 binds to SmCPS1, SmCPS5, and SmKSL1 gene promoters’ W-box elements, activating gene transcription and promoting tanshinone biosynthesis.^[49]^ Another member, SmWRKY40, binds to the W-box in the promoters of SmCPS1 and SmCPS5, inhibiting tanshinone synthesis.^[50]^ SmWRKY54, while increasing tanshinone production by binding to SmKSL, also interacts with genes in the salicylic acid signaling pathway through the W-box, enhancing drought resistance in Arabidopsis.^[51]^ SmWRKY61 has a strong regulatory effect on tanshinone accumulation, mainly by regulating the expression of genes in the MEP pathway (DXS2, CMK, and 3-hydroxy-3-methylglutaryl-CoA synthase 2) and downstream pathway (CPS, KSL, KSL2, CYP76AH1, and CYP76AK3), promoting tanshinone accumulation.^[52]^ SmWRKY34 negatively regulates tanshinone synthesis by directly regulating SmGGPPS.^[45]^

#### 3.3.6. Other transcription factors

The LBD family of TFs consists of an N-terminal LOB domain and a variable C-terminal, which can be divided into classes I and II.^[72]^ MeJA has a significant inducing effect on class II member SmLBD44, which inhibits tanshinone synthesis. Further research has shown that SmLBD44 inhibits the activity of SmKSL1, but SmJAZ1 can counteract the inhibitory effect of SmLBD44 on SmKSL1, indicating that SmLBD44 may be involved in the JA signaling pathway-mediated regulation of tanshinone synthesis.^[53]^

**Figure 2.**
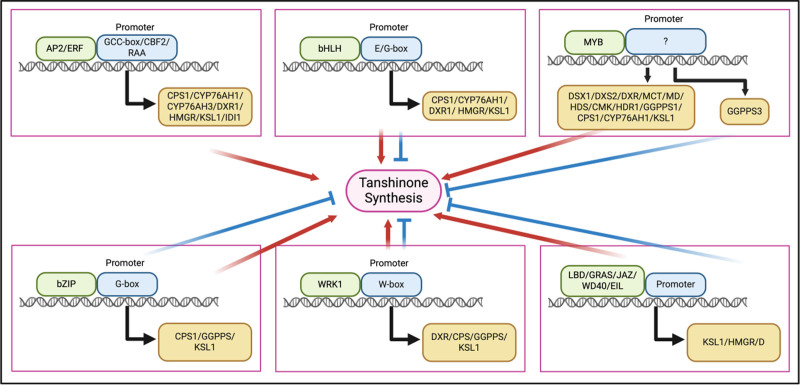
The regulatory role of transcription factors in tanshinone synthesis. AP2/ERF = APETALA2/ethylene response factor, bHLH = basic helix-loop-helix, bZIP = basic leucine zipper, CBF2 = C-repeat-binding transcription factor 2, DXR = 1-deoxy-D-xylulose 5-phosphate reductoisomerase, EIL = Ethylene-insensitive3-like, GCC = Guanylate cyclization C, GRAS = GAI-RGA-and-SCR, JAZ = Jasmonate ZIM-domain, LBD = Lateral Organ Boundaries Domain, MYB = myeloblastosis, RAA = Recombinase Aided Amplification, WRK1 = probable WRKY transcription factor protein 1.

GRAS proteins contain a less conserved N-terminal variable region and a conserved C-terminal GRAS domain. The typical GRAS domain includes 5 conserved sequence motifs: LHRI, VHIID, LHRII, PFYRE, and SAW.^[73]^ SmGRAS1/2/3/4/5 act as positive regulators affecting tanshinone synthesis, with different mechanisms of action. SmGRAS1/3/4/5 promote tanshinone synthesis by directly binding to the GARE-motif in the promoter of SmKSL1 and activating gene expression, while SmGRAS2 may regulate tanshinone synthesis through interaction with SmGRAS1^[54]^ (Fig. 2).

JAZ inhibitors are a class of repressor proteins in the JA pathway, responding to JA stimuli and interacting with other TFs to regulate their functions, constituting a key link in the JA signaling pathway regulation of secondary metabolite synthesis.^[74]^ Under MeJA treatment, overexpression of SmJAZ1/2/5/6/9 significantly increases tanshinone accumulation in hairy roots, while overexpression of SmJAZ3/4/8 reduces tanshinone accumulation. Additionally, SmJAZs form a complex regulatory network with SmMYC2a, SmMYC2b, SmMYB39, and SmPAP1, exhibiting multifunctionality, diversity, and redundancy in JA-induced tanshinone synthesis.^[27]^

In addition to the aforementioned TFs, overexpression of SmWD40-170 increases the content of tanshinone IIA and cryptotanshinone, significantly upregulating the expression levels of key enzyme genes SmDXS, SmHMGR, SmFPPS, SmGGPPS, SmCPS, and SmKSL.^[55]^ SmEIN3 (a key TF in the ethylene signaling pathway) positively regulates tanshinone synthesis by directly activating the expression of key enzyme genes such as SmHMGR and SmDXS2. Additionally, SmEIN3 interacts with SmMYC2 (a key TF in the JA signaling pathway), linking the ethylene signaling pathway with the JA signaling pathway to jointly regulate tanshinone synthesis.^[56]^ Current research on the involvement of WD40 and EIL family TFs in the biosynthesis of tanshinone compounds is limited, and their regulatory roles and mechanisms require further investigation.

## 4. Conclusion and prospect

This review has explored the intricate roles of TFs in the biosynthesis of tanshinones, highlighting both the complexity and the potential for targeted manipulation of these pathways. TFs are crucial in modulating the biosynthetic pathways of tanshinones by activating or repressing key genes involved in their production. Our discussion spanned several major TF families, including AP2/ERF, bHLH, MYB, bZIP, and WRKY, each playing unique roles in the regulation of the tanshinone biosynthesis.

One of the key insights derived from this review is the dualistic nature of TF regulation. While some TFs activate critical genes in the tanshinone biosynthesis pathway, others act as repressors, illustrating the fine balance required for optimizing tanshinone production. For example, the ERF and bHLH families positively regulate tanshinone synthesis by binding to gene promoters involved in precursor formation and subsequent enzymatic modifications. Conversely, certain members of the MYB and WRKY families can repress these pathways, adding another layer of complexity to the regulatory networks. However, while significant progress has been made in identifying and characterizing these TFs, gaps remain in fully understanding their cooperative interactions and the environmental stimuli that modulate their activity. For instance, although advances have been made in elucidating the effects of elicitors such as MeJA on TF activation, further research is needed to comprehensively map these signaling pathways and their influence on tanshinone production.

Looking forward, there are several promising avenues for research. One major challenge remains the full elucidation of the transcriptional networks governing tanshinone biosynthesis. Advances in omics technologies, particularly transcriptomics and proteomics, will be essential in mapping these networks in greater detail. These technologies can help identify new TFs involved in tanshinone biosynthesis, elucidate their regulatory mechanisms, and understand their interactions with other cellular components. Furthermore, future research should focus on employing advanced molecular techniques, such as clustered regularly interspaced short palindromic repeats/CRISPR-associated protein 9 gene editing and chromatin immunoprecipitation sequencing, to explore how TFs regulate gene expression in tanshinone biosynthesis. In-depth studies on protein-DNA interactions and epigenetic modifications will help elucidate the precise mechanisms by which these TFs modulate biosynthetic pathways. Additionally, validating these regulatory roles through in vivo experiments using genetically engineered plants will be essential to understanding how TFs influence tanshinone accumulation under natural conditions. Another promising research direction is the application of synthetic biology to reconstruct tanshinone biosynthesis in microbial or other plant systems. This approach could not only facilitate the production of tanshinones under controlled conditions but also enable the production of novel tanshinone derivatives with potentially enhanced pharmacological properties. Finally, integrating the knowledge from basic biological research into practical applications will be crucial. Enhancing the yield of tanshinones in *S miltiorrhiza* through genetic and metabolic engineering could significantly impact the pharmaceutical industry, providing more effective and potentially cheaper medicinal products.

In conclusion, while the study of TFs in tanshinone biosynthesis has advanced significantly, much remains to be understood. The ongoing development of molecular biology techniques and systems biology approaches holds great promise for unlocking the full potential of *S miltiorrhiza* as a medicinal plant and for advancing our understanding of plant secondary metabolism in general.

## Author contributions

**Writing – original draft:** Yanyun Pan.

**Visualization:** Jin Dai.

**Conceptualization:** Minwei Jin, Qiujun Zhou.

**Supervision:** Minwei Jin.

**Software:** Xiaoliang Jin.

**Writing – review & editing:** Jinjie Zhang.
